# Fetuin-A in Infants Born Small- or Large-for-Gestational-Age

**DOI:** 10.3389/fendo.2020.567955

**Published:** 2020-09-30

**Authors:** Wen-Juan Wang, Shufan Wang, Meng-Nan Yang, Yu Dong, Hua He, Fang Fang, Rong Huang, Xiao-Gang Yu, Guang-Hui Zhang, Xia Zhao, Tao Zheng, Xiao-Yi Huang, Jun Zhang, Fengxiu Ouyang, Zhong-Cheng Luo

**Affiliations:** ^1^Ministry of Education-Shanghai Key Laboratory of Children's Environmental Health, and Department of Pediatrics, Xinhua Hospital, Shanghai Jiao-Tong University School of Medicine, Shanghai, China; ^2^Department of Obstetrics and Gynecology, Faculty of Medicine, Dalla Lana School of Public Health, Lunenfeld-Tanenbaum Research Institute, Prosserman Center for Population Health Research, Mount Sinai Hospital, and Institute of Health Policy, Management and Evaluation, University of Toronto, Toronto, ON, Canada; ^3^Department of Clinical Assay Laboratory, Xinhua Hospital, Shanghai Jiao-Tong University School of Medicine, Shanghai, China; ^4^Department of Obstetrics and Gynecology, Xinhua Hospital, Shanghai Jiao-Tong University School of Medicine, Shanghai, China; ^5^Department of Pediatric, International Peace Maternity and Child Health Hospital, Shanghai Jiao-Tong University School of Medicine, Shanghai, China

**Keywords:** insulin-like growth factor, large-for-gestational-age, small-for-gestational-age, insulin, fetuin A

## Abstract

Fetuin-A is a multifunctional glycoprotein that has been implicated in insulin resistance and bone metabolism. We assessed whether fetuin-A is associated with poor or excessive fetal growth. In the Shanghai Birth Cohort, we conducted a nested case-control study of 60 trios of small-for-gestational-age (SGA, birth weight <10th percentile), optimal-for-gestational-age (OGA, 25–75th, the reference) and large-for-gestational-age (LGA, >90th percentile) infants matched by sex and gestational age. Cord plasma concentrations of fetuin-A and fetal growth factors [insulin, proinsulin, insulin-like growth factor (IGF)-I and IGF-II] were measured. Cord plasma fetuin-A concentrations were higher in SGA (809.4 ± 306.9 μg/ml, *P* = 0.026) and LGA (924.2 ± 375.9 μg/ml, *P* < 0.001) relative to OGA (680.7 ± 262.1 μg/ml) newborns, and were not correlated to insulin, proinsulin, IGF-I and IGF-II (all *P* > 0.2). Higher fetuin-A concentrations were associated with increased risks of SGA [OR = 1.67 (1.08–2.58) per SD increment, *P* = 0.024] and LGA [OR = 2.36 (1.53–3.66), *P* < 0.001]. Adjusting for maternal and neonatal characteristics and fetal growth factors, the elevated risk changed little for LGA [adjusted OR = 2.28 (1.29–4.01), *P* = 0.005], but became non-significant for SGA (*P* = 0.202). Our study is the first to demonstrate that fetuin-A may be involved in excessive fetal growth. This association is independent of fetal growth factors.

## Introduction

Fetuin-A or α2-HS-glycoprotein (AHSG) is a liver-derived glycoprotein, and has been implicated in insulin resistance and metabolic syndrome related disorders (1–4). Fetuin-A inhibits insulin receptor signaling by binding to insulin receptor tyrosine kinase (5, 6). AHSG knockout mice manifest improved insulin sensitivity (3). Human studies have associated elevated circulating fetuin-A concentrations with diabetes, obesity and non-alcoholic fatty liver disease in adults (2, 7).

Abnormal (poor or excessive) fetal growth is associated with elevated risks of metabolic syndrome related disorders in adulthood (8, 9). A variety of maternal and fetal elements may contribute to abnormal fetal growth (10, 11). Fetuin-A has been implicated in the regulation of bone growth (12). Animal studies showed that AHSG knockout mice had stunted femur growth (13). However, growth restricted pigs had higher plasma fetuin-A concentrations compared with normal sized littermate at birth (14), suggesting fetuin-A may have different implications for fetal growth across species.

Both insulin resistance and bone growth are relevant for fetal growth (15, 16), suggesting that fetuin-A may be implicated in abnormal fetal growth. A negative association has been observed between maternal circulating fetuin-A level and fetal growth (17, 18). Data are scarce concerning whether cord blood fetuin-A is associated with fetal growth. A small study reported no significant differences in cord serum fetuin-A concentrations between newborns with fetal growth restriction (*n* = 20) vs. normal birth weight (19), while another small study reported marked defects in the glycosylation of fetuin-A in small-for-gestation-age (*n* = 10) newborns (20). We are unaware of any study on cord blood fetuin-A concentration in excessive fetal growth. The aim of the present study was to evaluate whether cord blood fetuin-A concentration is associated with poor or excessive fetal growth.

## Materials and Methods

### Study Design, Population and Specimens

We performed a nested matched case-control study based on the recently described Shanghai Birth Cohort (SBC) (21). Briefly, the SBC is a prospective birth cohort study including 4,127 pregnant women in Shanghai, 2013–2016. Data on maternal, pregnancy and delivery characteristics were collected. Umbilical cord blood samples [in multiple tubes for serum (non-coagulant) and plasma (EDTA)] were collected in a standardized protocol by trained research staff immediately after delivery. Serum and plasma samples were obtained by centrifugation (centrifuge: BECKMAN COULTER Allegra X-15R, USA) at 4°C, 4,000 rpm for 10 min, and were stored in multiple aliquots at −80°C until assays. The study was approved by the research ethics committees of Xinhua Hospital (ref no. M2013-010) and all participating hospitals, Shanghai, China. Written informed consent was obtained from all study participants. The study adhered to the guidelines of the Declaration of Helsinki.

There were a total of 3,692 singleton live births with data available on birth weight and gestational age. Birth weight was measured by an electronic weighing device to the nearest gram. Gestational age (weeks) was calculated based on the date of last menstruation period (LMP) and confirmed by first trimester ultrasound dating. If the ultrasound dating was more than 2 weeks from the LMP-based estimate, the ultrasound dating-based gestational age was used. Infants were classified as small-for-gestational-age (SGA, <10th percentile), appropriate-for-gestational-age (AGA, between 10 and 90th percentile) and large-for-gestational-age (LGA, >90th percentile) according to the 2015 Chinese sex- and gestational age-specific birth weight standards (22). Among the AGA infants, we defined optimal-for-gestational-age (OGA) as birth weight between 25 and 75th percentiles, for the purpose of maximizing the contrasts to infants with poor (SGA) or excessive (LGA) fetal growth.

**Figure 1:**
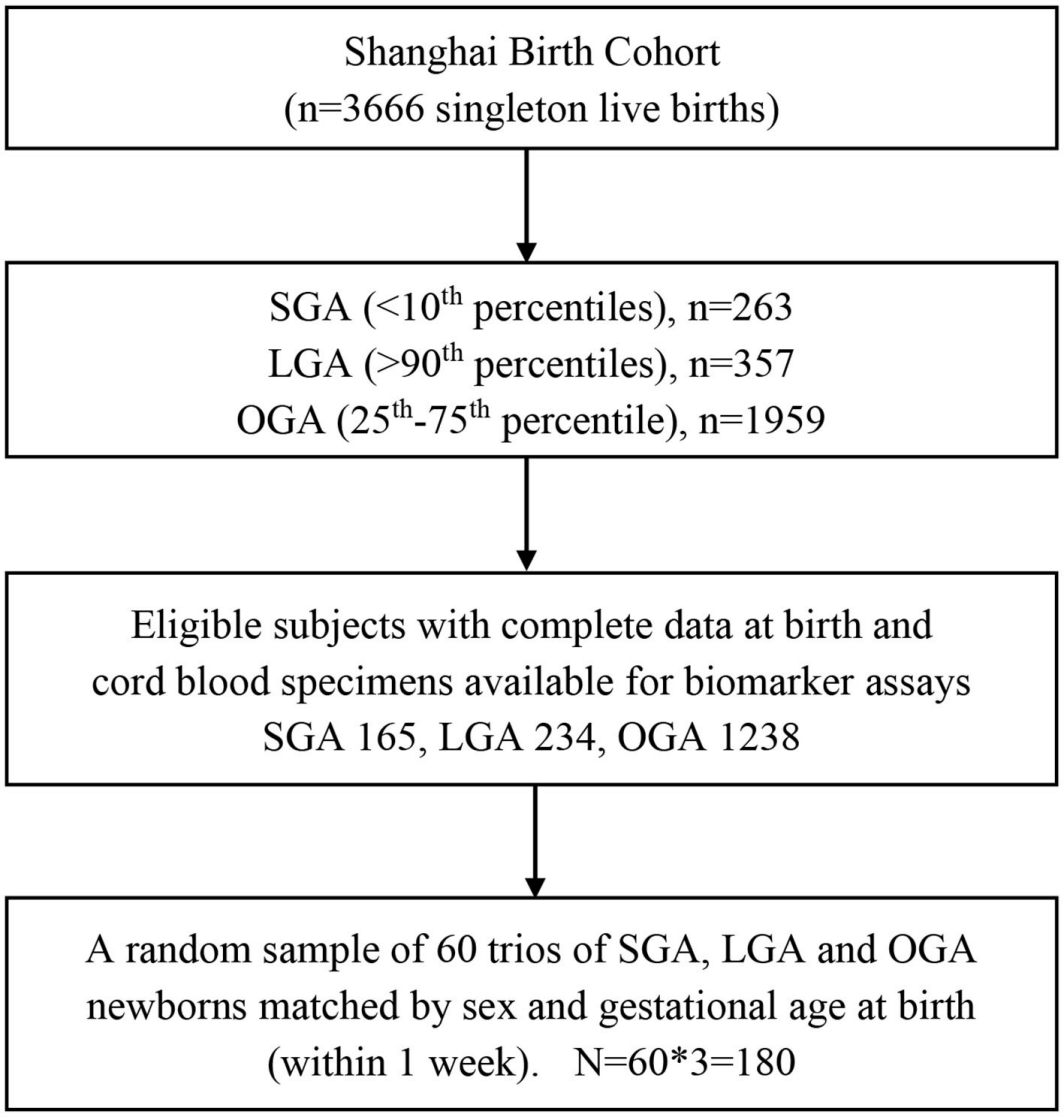
Flowchart in the selection of study subjects in a nested matched case control study of SGA, LGA, and OGA newborns in the Shanghai Birth Cohort. SGA, small-for-gestational-age (birth weight <10th percentile); LGA, large-for-gestational-age (>90th percentile); OGA, optimal-for-gestational-age (25–75th percentiles).

The present study is a random sample of 60 trios of SGA, OGA and LGA infants matched by sex (the same) and gestational age at birth (within 1 week). Eligible study subjects must meet all the following criteria: (1) Han ethnicity (the majority ethnic group, >98%); (2) maternal age 20–45 years; (3) natural conception; (4) singleton pregnancy; (5) free of maternal severe chronic disease or severe pregnancy complications (e.g., diabetes, heart disease, preeclampsia/eclampsia); (6) no birth defects; (7) 5-min Apgar score > = 7; (8) cord blood specimens available for biomarker assays. Therefore, the study sample included 180 singleton newborns (60 × 3). Their mothers were all non-smokers. Figure 1 presents the flowchart in the selection of study subjects. The study had a power of 85% to detect a 0.6 SD or greater difference in cord plasma fetuin-A concentration between SGA vs. OGA, or LGA vs. OGA infants, accounting for multiple tests.

### Biochemical Assays

Plasma fetuin-A (R&D system, Minnesota, USA), proinsulin (Mercodia, Uppsala, Sweden), and IGF-II (R&D system, MI, USA) were measured by enzyme-linked immunosorbent assay (ELISA) kits, and the absorbance was determined using a microplate spectrophotometer (Beckman CX7, USA). Serum insulin and insulin-like growth factor 1 (IGF-I) concentrations were detected by automated chemiluminescent assays (ADVIA Centaur and Immulite 2000, SIEMENS, Germany). The minimal detectable concentrations were 0.62 ng/ml for fetuin-A, 3.5 pmol/L for insulin, 1.7 pmol/L for proinsulin, 25 ng/ml for IGF-I and 1.88 pg/ml for IGF-II, respectively. The intra-assay and inter-assay coefficients of variation were in the ranges of 0.5–8.3% for fetuin-A, 2.0–6.5% for insulin and IGF-I, 0.34–5.0% for proinsulin, and 2.4–9.3% for IGF-II, respectively. We measured not only insulin but also proinsulin because both are positively associated with birth weight (23).

### Statistical Analysis

Continuous variable data were presented as Mean ± SD. Categorical variable data were presented as n (%). Biomarker data were log-transformed for I-tests and correlation analyses. Paired Student's *t*-test and Chi-squared test were used to evaluate the differences between two groups (SGA vs. OGA; LGA vs. OGA). Pearson partial correlation coefficients were calculated to evaluate the associations of cord blood fetuin-A with fetal growth factors adjusted for gestational age at delivery. Multinomial logistic regression models were fitted to assess the associations of cord blood fetuin-A with abnormal fetal growth (SGA, LGA) adjusted for maternal and neonatal characteristics. Z (standard deviation) scores of biomarker data were used in multinomial logistic regression models to facilitate the comparisons of effect sizes. There were no significant interactions between predictor variables affecting the primary effect estimates of interest. *P* < 0.025 was considered statistically significant in testing the primary hypothesis on the differences in fetuin-A concentrations between SGA vs. OGA, or LGA vs. OGA infants (Bonferroni correction for 2 tests).

Data analyses were performed using R, Version 3.5.1. Packages PPCOR and NNET were used in partial correlation and multinomial logistic regression analyses, respectively. The frequencies of missing values in co-variables were low (<7%). In the multinomial logistic regression analyses, multiple imputations were conducted using the MICE package in R. We created 25 datasets with imputations on missing data, and presented the results on the pooled regression coefficient statistics. We conducted sensitivity analysis to examine the multinomial logistic regression results without data imputations on missing values.

## Results

Maternal and neonatal characteristics are presented in Table 1. LGA newborns had higher maternal pre-pregnancy BMI, and were more frequently delivered by cesarean section than OGA newborns. As expected, mothers of LGA infants had higher blood glucose concentrations in the 75 g 2-h oral glucose tolerance tests (fasting, 1-h and 2-h) at 24–28 weeks of gestation. SGA infants had lower maternal pre-pregnancy BMI, but did not differ significantly from OGA infants in other characteristics.

**Table 1:** Maternal and neonatal characteristics in a matched study of SGA, OGA, and LGA singleton newborns in Shanghai Birth Cohort^*^.

	**OGA**	**SGA**	**LGA**	***P*^a^**	***P*^b^**
***N***	**60**	**60**	**60**		
**Maternal characteristics**					
Age, years	30.0 ± 3.5	29.4 ± 3.3	28.9 ± 3.5	0.324	0.080
>35	6 (10)	4 (6.7)	3 (5.0)	0.741	0.488
Education (university)	39 (65)	36 (60)	38 (63)	0.491	0.752
Drinking alcohol	8 (13)	1 (1.7)	2 (3.3)	0.037	0.180
Family history of diabetes	6 (10)	7 (12)	5 (8.3)	0.617	0.901
Pre-pregnancy BMI (kg/m^2^)	21.2 ± 2.7	20.1 ± 2.5	22.4 ± 2.6	0.058	**0.009**
BMI group				**0.005**	**0.001**
Underweight (<18.5)	8 (13)	15 (23)	1 (2)		
Normal weight (18.5–24.0)	45 (75)	31 (52)	36 (60)		
Overweight (≥24.0)	6 (10)	4 (6.7)	12 (20)		
Primiparity	47 (78)	55 (92)	49 (82)	0.074	0.820
**75 g OGTT (mmol/L)**					
Fasting	4.34 ± 0.40	4.42 ± 0.38	4.61 ± 0.39	0.323	**<0.001**
1-h	7.36 ± 1.4	7.77 ± 1.7	8.22 ± 1.5	0.309	**0.002**
2-h	6.28 ± 1.0	6.53 ± 1.6	6.77 ± 1.2	0.252	0.069
HbA1C (%)	5.05 ± 0.32	4.94 ± 0.32	5.09 ± 0.35	0.244	0.561
**Neonatal characteristics**					
C-section delivery	12 (20)	17 (28)	35 (58)	0.333	**<0.001**
Sex, male	33 (55)	33 (55)	33 (55)	1.00	1.00
Gestational age (weeks)	39.6 ± 1.1	39.5 ± 1.2	39.6 ± 1.2	0.130	0.825
Birth weight (g)	3,372 ± 264	2,674 ± 293	4,162 ± 351	**<0.001**	**<0.001**
*z* score	0.13 ± 0.65	−1.63 ± 0.68	2.10 ± 0.76	**<0.001**	**<0.001**
Birth length (cm)	49.8 ± 1.2	48.7 ± 1.5	51.2 ± 1.1	**<0.001**	**<0.001**
z score	−0.15 ± 0.98	−1.08 ± 1.30	1.21 ± 1.00	**<0.001**	**<0.001**

* *Data presented are Mean ± SD or n (%). The study subjects were 60 trios of SGA, OGA, and LGA newborn infants matched by sex and gestational age (weeks) at delivery; all mothers were non-smokers*.

*SGA, small-for-gestational-age (birth weight <10th percentile); OGA, optimal-for-gestational-age (25–75th h percentiles); LGA, large-for-gestational-age (>90th percentile); OGTT, oral glucose tolerance test (at 24–28 weeks of gestation)*.

a *P values comparing SGA vs. OGA groups*.

b *P values comparing LGA vs. OGA groups in paired t-tests for continuous variables or chi-square tests for categorical variables*.

*P values in bold, p < 0.025*.

**Figure 2:**
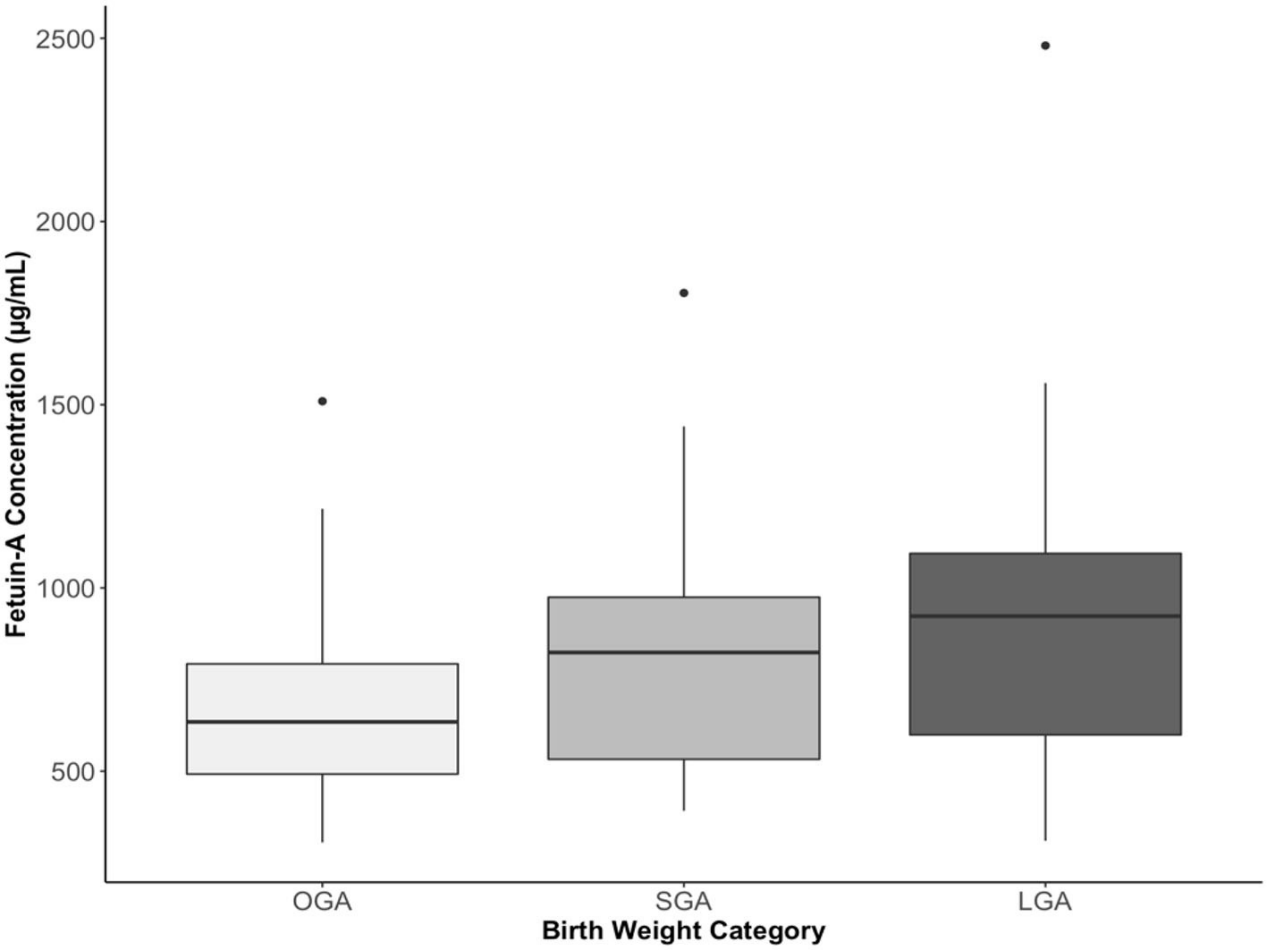
Cord plasma fetuin-A concentrations in small-, optimal-, or large-for-gestational-age (SGA, OGA, LGA) infants. The boxes represent the medians and interquartile ranges, the dots represent the maximal values. *P* = 0.024 comparing SGA vs. OGA, and *P* < 0.001 comparing LGA vs. OGA.

Compared to OGA infants, cord plasma fetuin-A concentrations were significantly higher in both SGA (*P* = 0.024) and LGA (*P* < 0.001) infants (Table 2 and Figure 2). For all fetal growth factors (insulin, IGF-I, and IGF-II), cord blood concentrations were the lowest in SGA infants, and the highest in LGA infants. IGF-I and proinsulin concentrations were significantly lower in SGA infants, and higher in LGA infants than in OGA infants. Insulin concentrations were significantly lower in SGA infants.

**Table 2:** Cord blood concentrations of fetuin-A and fetal growth factors in SGA, OGA, and LGA infants.

	**OGA**	**SGA**	**LGA**	***P*^a^**	***P*^b^**
Fetuin-A (μg/mL)	680.7 ± 262.1	809.4 ± 306.9	924.2 ± 375.9	**0.024**	**<0.001**
Insulin (pmol/L)	35.3 ± 34.9	21.7 ± 16.3	38.7 ± 31.8	0.027	0.407
Proinsulin (pmol/L)	19.9 ± 16.6	12.3 ± 6.30	32.2 ± 28.2	**0.001**	**0.010**
IGF-1 (ng/mL)	65.9 ± 27.4	48.5 ± 17.3	86.5 ± 26.8	**<0.001**	**<0.001**
IGF-2 (ng/mL)	188.2 ± 29.8	181.2 ± 26.6	194.9 ± 29.0	0.311	0.130

*Data presented are Mean ± SD*.

*SGA, small-for-gestational-age (birth weight <10th percentile); OGA, optimal-for-gestational-age (25–75th h percentiles); LGA, large-for-gestational-age (>90th percentile)*.

a *P values comparing SGA and OGA groups*;

b *P values comparing LGA and OGA groups in paired t-tests of log-transformed biomarker data*.

*P values in bold, p < 0.025*.

Adjusting for gestational age at delivery/specimen sampling, cord blood Fetuin-A was not correlated with insulin, proinsulin, IGF-I and IGF-II or maternal fasting blood glucose (all *P* > 0.2, Table 3).

**Table 3:** Pearson partial correlation coefficients ^*^ of cord blood fetuin-A with fetal growth factors and maternal fasting glucose (24–28 weeks of gestation).

	***r***	***P***
IGF-I	0.023	0.772
IGF-II	0.090	0.237
Insulin	0.021	0.779
Proinsulin	−0.006	0.939
Maternal fasting glucose	0.045	0.572

*IGF, insulin-like growth factor*.

* *Data presented are Pearson partial correlations in log-transformed data adjusting for gestational age at birth/blood sampling. The correlations were similar for small-for-gestational-age, optimal-for-gestational-age, and large-for-gestational-age infants; therefore, the results for the total sample were presented*.

Without any adjustment, higher cord plasma fetuin-A concentrations were associated with increased odds of both LGA and SGA (Table 4). Adjusted for maternal and neonatal characteristics, the OR for SGA became no longer statistically significant, while the OR remained highly significant for LGA (OR = 2.42, 95% CI 1.47–3.97). Among the fetal growth factors, higher cord blood IGF-I and proinsulin concentrations were strongly associated with a lower odds of SGA and a higher odds of LGA. Higher cord blood insulin concentrations were associated with a lower odds of SGA (OR = 0.45, 95% CI 0.25–0.81). Cord plasma IGF-II was not associated with SGA or LGA.

**Table 4:** Associations of cord blood fetuin-A and fetal growth factors (insulin, proinsulin, IGF-1, IGF-2) with the risks of SGA and LGA.

	**Crude OR (95% CI)**	***P***	^*^Adjusted OR (95% CI)	***P***
**Fetuin-A**				
SGA	1.67 (1.08–2.58)	**0.024**	1.46 (0.92–2.32)	0.110
LGA	2.36 (1.53–3.66)	**<0.001**	2.42 (1.47–3.97)	**<0.001**
**IGF-I**				
SGA	0.30 (0.16–0.55)	**<0.001**	0.34 (0.17–0.68)	**0.003**
LGA	2.34 (1.50–3.65)	**<0.001**	2.49 (1.45–4.27)	**0.001**
**IGF-II**				
SGA	0.77 (0.52–1.14)	0.197	0.83 (0.54–1.28)	0.406
LGA	1.25 (0.87–1.80)	0.224	1.43 (0.91–2.23)	0.120
**Insulin**				
SGA	0.48 (0.28–0.82)	**0.008**	0.45 (0.25–0.81)	**0.009**
LGA	1.10 (0.79–1.52)	0.578	0.92 (0.63–1.35)	0.677
**Proinsulin**				
SGA	0.12 (0.04–0.42)	**0.001**	0.15 (0.04–0.51)	**0.003**
LGA	1.84 (1.15–2.93)	**0.011**	1.66 (1.05–2.62)	**0.033**

*SGA, small-for-gestational-age (<10th percentile); LGA, large-for-gestational-age (>90th percentile)*.

* *The odds ratio per SD increase in each biomarker from multinomial logistic regression models adjusted for maternal fasting glucose, pre-pregnancy BMI, primiparity and C-section; other maternal and neonatal characteristic co-variables were excluded since they were not significant (all p > 0.2) and did not affect the comparisons*.

*P values in bold, p < 0.025*.

In the fully adjusted model including maternal, neonatal characteristics and fetal growth factors (Table 5), the OR for fetuin-A in association with LGA or SGA changed little compared to the OR adjusted for maternal and neonatal characteristics only (Table 4). The elevated risk after the adjustment remained significant for LGA (OR = 2.28, *P* = 0.005), and became not statistically significant for SGA (OR = 1.41, *P* = 0.202). Similar findings were observed in the multinomial logistic regression analyses without imputations for missing data (results not shown).

**Table 5:** Associations of maternal characteristics, cord plasma fetuin-A and fetal growth factors with the risks of SGA and LGA in the fully adjusted models^*^.

	**SGA**		**LGA**	
	**OR (95% CI)**	***P*****-value**	**OR (95% CI)**	***P*****-value**
**Pre-pregnancy BMI**				
Normal weight	reference		reference	
Overweight (>24.0)	1.49 (0.30–7.32)	0.625	3.44 (0.79–14.93)	0.102
Underweight (<18.5)	1.88 (0.60–5.89)	0.279	0.26 (0.03–2.63)	0.255
Primiparity	1.52 (0.39–5.92)	0.550	1.84 (0.48–6.99)	0.375
C-section	2.34 (0.81–6.80)	0.120	4.83 (1.63–14.25)	**0.005**
OGTT fasting glucose	1.47 (0.91–2.40)	0.121	2.16 (1.19–3.93)	**0.012**
**Cord plasma**				
Fetuin-A	1.41 (0.83–2.37)	0.202	2.28 (1.29–4.01)	**0.005**
IGF-1	0.41 (0.19–0.88)	**0.023**	2.74 (1.40–5.35)	**0.004**
IGF-2	0.76 (0.46–1.25)	0.286	0.97 (0.57–1.65)	0.902
Insulin	0.51 (0.23–1.14)	0.103	0.57 (0.31–1.05)	0.073
Proinsulin	0.26 (0.06–1.03)	0.057	2.07 (1.02–4.19)	0.046

* *Data presented are the effect estimates (OR) in the final models including pre-pregnancy BMI, maternal fasting glucose, primiparity, C-section and cord blood fetuin-A, IGF-1, IGF-2, insulin and proinsulin; other maternal and neonatal characteristic variables were excluded since they were not associated with SGA or LGA (p > 0.2) and did not affect the comparisons. The effect estimates are for per SD increase in each biomarker*.

*P values in bold, p < 0.025*.

## Discussion

We observed elevated cord blood fetuin-A concentrations in infants with poor (SGA) or excessive (LGA) fetal growth relative to infants with optimal fetal growth (OGA). Higher cord blood fetuin-A concentrations were associated with an increased risk of LGA independent of fetal growth factors.

Our study is the first to report a strong positive association between cord blood fetuin-A and excessive fetal growth. The association is independent of IGF-I, IGF-II, proinsulin and insulin, suggesting it may be mediated by pathways other than fetal growth factors. Our data confirmed that cord blood IGF-I and proinsulin concentrations are strongly associated with fetal growth (23, 24).

We observed higher cord blood fetuin-A concentrations in SGA vs. OGA infants, but the adjusted OR became not statistically significant, suggesting that the association may be an artifact of confounding factors. Similarly, a small study reported that fetuin-A concentrations were not different in infants with fetal growth restriction (*n* = 20) compared to normal birth weight infants (19). It should be cautioned that both our and previous studies were not powered to detect relatively small differences. It is unclear whether the higher cord blood fetuin-A concentrations in SGA infants are related to their increased insulin resistance in childhood (25). Long-term follow-up studies are required to address this question.

We are unaware of any research data on cord blood fetuin-A concentration in excessive fetal growth. We observed that LGA - an indicator of excessive fetal growth, was associated with substantially elevated cord blood fetuin-A concentrations. Interestingly, a study in prepubertal children observed higher fetuin-A concentrations in overweight/obese relative to underweight/normal weight children (4), consistent with our finding to some extent but at a different life stage. Moreover, studies in children and adults have associated elevated circulating fetuin-A concentrations with obesity and other metabolic disorders (26, 27). We might speculate that elevated fetuin-A concentrations in LGA infants may play a role in early life development of the vulnerability to metabolic syndrome related disorders.

We could not determine whether the elevated fetuin-A concentration in LGA infants is a cause or consequence of excessive fetal growth. A previous study using bidirectional Mendelian randomization analysis suggests that AHSG (the gene coding fetuin-A) is casually related to BMI (28), lending some support in fetuin-A's role in the development of fetal overgrowth. The pathways relating fetuin-A to fetal growth are unknown. Studies have linked fetuin-A with insulin resistance (4). The production of pro-inflammatory cytokines as well as the mobilization of free fatty acids via toll-like-receptor-4 (1, 29). Some nutrients such as curcumin and niacin have been associated with decreased serum fetuin-A concentrations (30, 31). It remains to be explored whether these nutrients may be related to the association between fetuin-A and LGA.

The matched study is a powerful design to detect differences in cord blood biomarkers between groups. All study subjects are Chinese Han ethnicity, eliminating the potential confounding effect of ethnic difference in fetal growth. We used OGA (25–75th percentiles) rather than AGA (10–90th percentiles) infants as the comparison (control) group. This study approach may be more powerful in identifying the differences between infants with extreme (poor or excessive) vs. normal fetal growth due to stronger contrasts than a study using AGA as the comparison group (24). A main limitation is the lack of data on fetuin-A isoforms. We did not have the data on glycosylation and/or phosphorylation isoforms of fetuin-A. Future studies may further explore whether there are elevations in specific isoforms of cord blood fetuin-A in LGA infants. Our study was powered to detect moderate to large differences (>0.6 SD), but not powered to detect small differences. This is an observational study, and causality could not be affirmed. Reverse causality could not be ruled out. The study sample was restricted to Chinese infants. More studies in other ethnic groups/populations are required to confirm the generalizability of the study findings.

In conclusion, cord blood fetuin-A concentrations were elevated in SGA and LGA infants. Fetuin-A may be involved in excessive fetal growth independent of fetal growth factors (insulin, IGF-I and IGF-II).

## Data Availability Statement

The datasets presented in this article are not readily available because Access to the de-identified participant research data must be approved by the research ethics board on a case-by-case basis, please contact the corresponding author for assistance in data access request. Requests to access the datasets should be directed to Zhong-Cheng Luo, zcluo@lunenfeld.ca; Fengxiu Ouyang, ouyangfengxiu@xinhuamed.com.cn.

## Ethics Statement

The Institutional Review Board of Xinhua Hospital, Shanghai Jiao-Tong University School of Medicine approved this study (ref no. M2013-010, date of approval: August 23, 2013). The patients/participants provided their written informed consent to participate in this study.

## Author Contributions

Z-CL, JZ, and FO conceived the study with inputs from all co-authors. W-JW, M-NY, YD, HH, FF, RH, X-GY, G-HZ, XZ, TZ, X-YH, JZ, FO, and Z-CL contributed to the acquisition of research data. W-JW and SW conducted the literature review, data analysis, and drafted the manuscript. Z-CL is the guarantor of this work, has full access to all the data in the study, and takes responsibility for the integrity of the data and the accuracy of the data analysis. All authors contributed in revising the article critically for important intellectual content and approved the final version for publication.

## Conflict of Interest

The authors declare that the research was conducted in the absence of any commercial or financial relationships that could be construed as a potential conflict of interest.

## Abbreviations

LGA: large-for-gestational-age
OGA: optimal-for-gestational-age
SGA: small-for-gestational-age
IGF: insulin-like growth factor
OR: odds ratio.
